# Sarcocrassocolides M–O, Bioactive Cembranoids from the Dongsha Atoll Soft Coral *Sarcophyton crassocaule*

**DOI:** 10.3390/md10030617

**Published:** 2012-03-08

**Authors:** Wan-Yu Lin, Yi Lu, Bo-Wei Chen, Chiung-Yao Huang, Jui-Hsin Su, Zhi-Hong Wen, Chang-Feng Dai, Yao-Haur Kuo, Jyh-Horng Sheu

**Affiliations:** 1 Department of Marine Biotechnology and Resources, National Sun Yat-sen University, Kaohsiung 804, Taiwan; Email: lemotylin@gmail.com (W.-Y.L.); snakefoot5052@gmail.com (Y.L.); a6152761@yahoo.com.tw (B.-W.C.); betty8575@yahoo.com.tw (C.-Y.H.); wzh@mail.nsysu.edu.tw (Z.-H.W.); 2 National Museum of Marine Biology & Aquarium, Pingtung 944, Taiwan; Email: x2219@nmmba.gov.tw; 3 Graduate Institute of Marine Biotechnology, National Dong Hwa University, Pingtung 944, Taiwan; 4 Institute of Oceanography, National Taiwan University, Taipei 112, Taiwan; Email: corallab@ntu.edu.tw; 5 National Research Institute of Chinese Medicine, Taipei 112, Taiwan; Email: kuoyh@nricm.edu.tw; 6 Asia-Pacific Ocean Research Center, National Sun Yat-sen University, Kaohsiung 804, Taiwan

**Keywords:** soft coral, *Sarcophytoncrassocaule*, cytotoxic activity, anti-inflammatory activity

## Abstract

Three new cembranoids, sarcocrassocolides M–O (**1**–**3**), have been isolated from the soft coral *Sarcophyton crassocaule*. The structures of the metabolites were determined by extensive spectroscopic analysis. Compounds **1**–**3** were shown to exhibit moderate cytotoxicity toward a limited panel of cancer cell lines and display significant *in vitro* anti-inflammatory activity in LPS-stimulated RAW264.7 macrophage cells by inhibiting the expression of the iNOS protein.

## 1. Introduction

**Chart 1:**
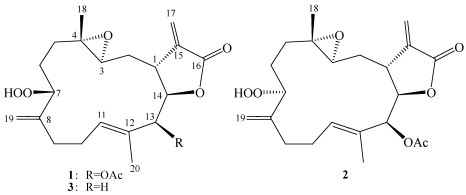
Structures of metabolites **1**–**3**.

Cembrane-type compounds have been found to be the important diterpenoidal constituents in marine coelenterates [1,2,3,4,5,6,7,8]. In the investigation of the bioactive metabolites from soft corals of Taiwanese waters, many bioactive cembranoids have been isolated from octocorals (Alcyonaceae) belonging to the genera *Sinularia* [9,10,11,12], *Lobophytum* [13,14], *Sarcophyton* [15,16,17,18,19,20] and *Pachyclavularia* [21]. Some of these metabolites have been shown to exhibit cytotoxic activity against the growth of various cancer cell lines [9,11,14,15,16,17,18,19,20,21], and/or anti-inflammatory activity [10,14]. Our recent study of the chemical constituents of the Dongsha Atoll soft coral *Sarcophyton crassocaule* [22,23] has yielded cembranoids sarcocrassocolides A–L, of which some were found to exhibit significant cytotoxic and anti-inflammatory activities. Our continuing chemical investigation on the same collection of this organism, with the aim of discovering other biologically active natural products, again led to the isolation of three new cembranoids, sarcrocrassocolides M–O (**1**–**3**) (Chart 1). The structures of **1**–**3** were established by extensive spectroscopic analysis, including careful examination of 2D NMR (^1^H–^1^H COSY, HMQC, HMBC and NOESY) correlations. The cytotoxicity of compounds **1**–**3** against human breast carcinoma (MCF-7), human colon carcinoma (WiDr), human laryngeal carcinoma (HEp-2) and human medulloblastoma (Daoy) cell lines was studied, and the ability of **1**–**3** to inhibit the up-regulation of pro-inflammatory iNOS (inducible nitric oxide synthase) and COX-2 (cyclooxygenase-2) proteins in LPS (lipopolysaccharide)-stimulated RAW264.7 macrophage cells was also examined. It was found that compounds **1**–**3** were cytotoxic towards the above cancer cells; **2** being the most cytotoxic. Compounds **1**–**3** were found to significantly inhibit the expression of iNOS protein.

## 2. Results and Discussion

**Figure 1:**
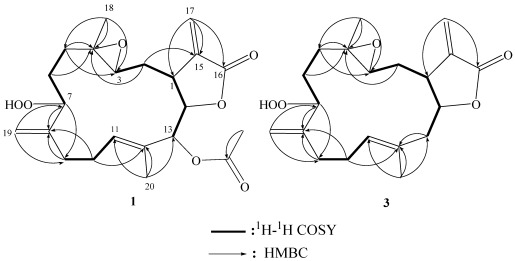
^1^H–^1^H COSY and HMBC correlations for **1** and **3**.

**Figure 2:**
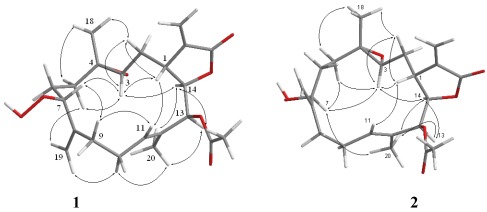
Key NOESY correlations for **1** and **2**.

The HRESIMS (*m/z* 429.1892 [M + Na]^+^) of sarcrocrassocolide M (**1**) established the molecular formula C_22_H_30_O_7_, appropriate for eight degrees of unsaturation, and the IR spectrum revealed the presence of lactonic carbonyl (1757 cm^−1^) group. The ^13^C NMR and DEPT (Table 1) spectroscopic data showed signals of three methyls (including one acetate methyl), five sp^3^ methylenes, two sp^2^ methylenes, five sp^3^ methines (including four oxymethines), one sp^2^ methines, one sp^3^ and five sp^2^ quaternary carbons (including two ester carbonyls). The NMR signals (Table 1) observed at δ_C_ 169.1 (C), 139.3 (C), 121.6 (CH_2_), 81.4 (CH), and 37.4 (CH), and δ_H_ 6.28, 5.63 (each, 1H, d, *J* = 2.0 Hz), 4.61 (1H, t, *J *= 2.5 Hz), and 3.07 (1H, dt, *J *= 11.5, 2.5 Hz) showed the presence of an α-methylene-γ-lactonic group by comparing with similar NMR data of known cembranoids with the same five-membered lactone ring [22,23]. Signals resonating at δ_C_ 60.1 (C), 60.0 (CH) and δ_H_ 2.53 (1H, dd, *J *= 7.0, 4.0 Hz) revealed the presence of a trisubstituted epoxide. One trisubstituted and one 1,1-disubstituted double bond were also identified from NMR signals appearing at δ_C_ 129.4 (C), 128.1 (CH), and δ_H_ 5.46 (1H, dd, *J *= 7.0, 5.5 Hz), and at δ_C_ 113.5 (CH_2_), 146.6 (C), δ_H_ 5.16 and 5.12 (1H, s, each), respectively. In the ^1^H–^1^H COSY spectrum, it was possible to identify three different structural units, which were assembled with the assistance of an HMBC experiment. Key HMBC correlations of H_3_-18 to C-3, C-4 and C-5; H_2_-19 to C-7, C-8 and C-9; H_3_-20 to C-11, C-12 and C-13 and H_2_-17 to C-1, C-15 and C-16 permitted the establishment of the carbon skeleton (Figure 1). Furthermore, the acetoxy group positioned at C-13 was confirmed from the HMBC correlations of the methyl protons of an acetate (δ_H_ 2.02) to the ester carbonyl carbon at δ_C_ 169.2 and the oxymethine signal at 77.4 (C-13, CH). The ^13^C NMR signals at δ_C_ 87.1 (CH) and HRESIMS showed the presence of a hydroperoxy group at a methine carbon C-7 [9]. On the basis of the above analysis, the planar structure of **1** was established unambiguously. The relative structure of **1** was elucidated by the analysis of NOE correlations, as shown in Figure 2. The NOE interaction of H-1 (δ 3.07) with H-2β (δ 1.71), H-3 (δ 2.57) and H-11 (δ 5.46) revealed the β-orientation of H-1 and H-3. H-3 did not exhibit NOE correlation with H_3_-18 (δ 1.32, s) instead it correlated with one proton of H_2_-5, reflecting the *trans *stereochemistry of 3,4-epoxide. The proton H-7 (δ 4.30) showed NOE interactions with H-3, and both H-7 and H-11 had NOE correlations with one proton of H_2_-9 (δ 2.14). Thus, H-7 was placed on the β-face. The *E *geometry of the trisubstituted double bond at C-11 and C-12 was assigned from the NOE correlation of H_3_-20 (δ 1.76) with one proton of H_2_-10 (δ 2.40), but not with the olefinic proton H-11, in addition to the upper field chemical shift of C-20 (δ 14.8). H-14 (δ 4.61) exhibited NOE correlations with both H-13 (δ 5.40) and H_3_-20, but not with H-1, indicating the α-orientation of both H-13 and H-14. These results, together with other detailed NOE correlations of **1** (Figure 2), unambiguously established the structure of sarcocrassocolide M, as shown in formula **1**. Therefore, the relative structure of compound **1** was determined.

**Table 1:** NMR spectropic data for sarcrocrassocolides M–O (**1**–**3**).

Position	Sarcrocrassocolide M (1)		Sarcrocrassocolide N (2)		Sarcrocrassocolide O (3)	
	δ_C_, mult.*^a^*	δ_H_ (*J* in Hz)*^ b^*	δ_C_, mult.*^a^*	δ_H_ (*J* in Hz)*^b^*	δ_C_, mult.*^c^*	δ_H_ (*J* in Hz)*^ d^*
1	37.4, CH	3.07, dt (11.5, 2.5)	37.5, CH	3.07, brd (11.5)	41.2, CH	2.78, ddd (11.2, 4.8, 2.8)
2	34.8, CH_2_	1.87, ddd (14.5, 11.5, 4.0)	35.1, CH_2_	1.86, ddd (15.5, 11.5, 6.0)	33.2, CH_2_	1.97, m
		1.71, m		1.71, ddd (15.5, 6.0, 2.0)		1.64, m
3	60.0, CH	2.53, dd (7.0, 4.0)	59.9, CH	2.59, t (6.0)	59.8, CH	2.63, dd (8.8, 4.6)
4	60.1, C		60.9, C		60.8, C	
5	32.8, CH_2_	1.98, ddd (14.0, 7.0, 3.5)	32.9, CH_2_	1.96, m	32.6, CH_2_	1.96, m
		1.46, dt (14.0, 7.0)		1.42, m		1.39, m
6	26.2, CH_2_	1.72, m	28.6, CH_2_	1.62, m	26.4, CH_2_	1.78, m
		1.58, m		1.43, m		1.52, m
7	87.1, CH	4.30, t (5.5)	86.1, CH	4.38, t (5.0)	86.6, CH	4.38, t (5.2)
8	146.6, C		147.9, C		146.3, C	
9	32.5, CH_2_	2.43, m	31.9, CH_2_	2.47, m	32.5, CH_2_	2.29, m
		2.14, ddd (14.5, 8.0, 4.0)		2.01, m		2.21, m
10	26.1, CH_2_	2.40, m	25.5, CH_2_	2.33, m	26.0, CH_2_	2.37, m
		2.29, m				2.23, m
11	128.1, CH	5.46, dd (7,0, 5.5)	127.4, CH	5.41, t (7.5)	128.8, CH	5.26, t (6.8)
12	129.4, C		129.4, C		130.5, C	
13	77.4, CH	5.40, s	77.4, CH	5.39, s	44.9, CH_2_	2.58, brd (14.4)
						2.32, dd (14.4, 8.0)
14	81.4, CH	4.61, t (2.5)	81.7, CH	4.62, s	82.1, CH	4.47, dt (8.0, 4.0)
15	139.3, C		139.3, C		138.8, C	
16	169.1, C		169.1, C		169.4, C	
17	121.6, CH_2_	6.28, d (2.0)	122.0, CH_2_	6.30, d (2.0)	122.8, CH_2_	6.32, d (2.4)
		5.63, d (2.0)		5.64, d (2.0)		5.62, d (2.4)
18	17.5, CH_3_	1.32, s	17.7, CH_3_	1.29, s	17.0, CH_3_	1.33, s
19	113.5, CH_2_	5.16, s	111.9, CH_2_	5.09, s	113.8, CH_2_	5.17, s
		5.12, s		5.08, s		5.12, s
20	14.8, CH_3_	1.76, s	15.1, CH_3_	1.78, s	17.3, CH_3_	1.70, s
OAc	20.7, CH_3_	2.02, s	20.7, CH_3_	2.03, s		
	169.2, C		169.2, C			

*^a^* Spectra recorded at 125 MHz in CDCl_3_; *^b^* Spectra recorded at 500 MHz in CDCl_3_; *^c^* Spectra recorded at 100 MHz in CDCl_3_; *^ d^* Spectra recorded at 400 MHz in CDCl_3_.

Compound **2** possessed the same molecular formula (C_22_H_30_O_7_) as that of **1**, as revealed from HRESIMS. Furthermore, it was found that the NMR spectroscopic data of **2** (Table 1) were similar to those of **1**. Analysis of the 2D NMR (^1^H–^1^H COSY, HMQC, and HMBC) correlations revealed that compound **2** possesses the same planar structure as that of **1**. From the NOESY spectrum, it was found that H-7 (δ 4.38) showed a weak NOE interaction with H_3_-20 (1.78), but not with H-11 (δ 5.41), revealing the α-orientation of H-7. Further analysis of other NOE interactions revealed that **2** possessed the same relative configurations at C-1, C-3, C-4, C-13 and C-14, as those of **1** (Figure 2). Therefore, **2** was found to be the C-7 epimer of **1**.

**Figure 3:**
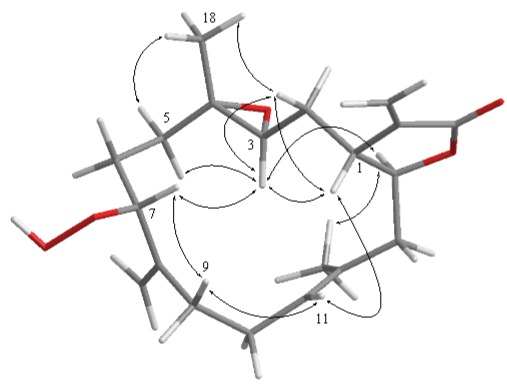
Key NOESY correlations for **3**.

Compound **3** was shown by HRESIMS to possess the molecular formula C_20_H_28_O_5_ (*m*/*z *371.1835 [M + Na]^+^). The IR spectrum of **3** also revealed the presence of lactonic carbonyl (1752 cm^−1^) group. Comparison of the ^1^H and ^13^C NMR data (Table 1) of compounds **1** and **3** showed that the structure of **3** should be very close to that of **1,** with the exception of signals assigned to C-13, where an acetoxymethine (δ_H_ 5.40, 1H, s; δ_C_ 77.4) in **1** was replaced by a methylene (δ_H_ 2.58, 1H, brd, *J* = 14.4 Hz, δ_H_ 2.32, 1H, dd, *J* = 14.4, 8.0 Hz; δ_C_ 44.9) in **3**. The planar structure of **3** was elucidated by analyzing the ^1^H–^1^H COSY and HMBC correlations (Figure 1). The relative stereochemistry of **3** was confirmed from the key NOESY correlations (Figure 3), and the structure of sarcocrassocolide O, as shown in formula **3**, was established unambiguously. Thus, **3** is the 13-deacetoxy derivative of **1**.

**Chart 2:**
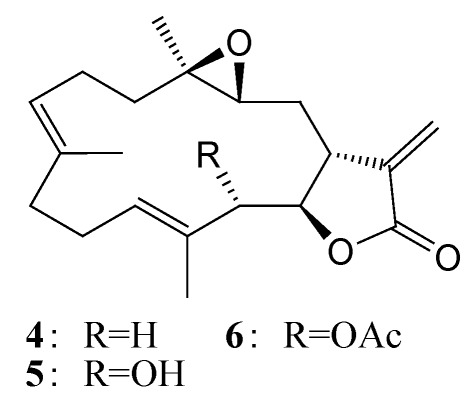
Structures of compounds **4**–**6**.

Similar to sarcocrassocolides F–L [22], **1**–**3** should be the oxidized products of the related 3α,4α-epoxycembranolides with 7,8-olefinic group, although we have not yet discovered the similar oxidation from cembranolides possessing 7,8-double bond and 3β,4β-epoxide, such as sarcocrassolide (**4**), sinularolide E (**5**), and 13-acetoxysarcocrassolide (**6**) (Chart 2), which were isolated by our previous study [24,25].

**Figure 4:**
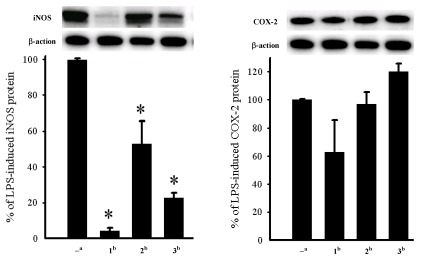
Effect of compounds **1**–**3** on inducible nitric oxide synthetase (iNOS) and cyclooxygenase-2 (COX-2) proteins expression of RAW264.7 macrophage cells by immunoblot analysis.(**A**) Immunoblots of iNOS and β-actin; (**B**) Immunoblots of COX-2 and β-actin. The values are mean ± SEM (*n *= 6). Relative intensity of the lipopolysaccharide (LPS) alone stimulated group was taken as 100%. * Significantly different from LPS alone stimulated group (*P < 0.05). *^a^* stimulated with LPS; *^b^* stimulated with LPS in the presence of **1**–**3** (10 μM).

The cytotoxicity of compounds **1**–**3** against the proliferation of a limited panel of cancer cell lines, including Daoy, HEp-2, MCF-7 and WiDr carcinoma cell lines was evaluated. The results (Table 2) showed that all compounds **1**–**3** were found to exhibit cytotoxicity against all or part of the above carcinoma cell lines**.** In this assay, the *in vitro *anti-inflammatory effects of compounds **1**–**3** were also tested. The inhibition of LPS-induced up-regulation of pro-inflammatory proteins, iNOS and COX-2 in RAW264.7 macrophage cells was measured by immunoblot analysis (Figure 4). At a concentration of 10 μM, compounds **1**–**3** were found to significantly reduce the levels of iNOS protein to 4.2 ± 1.6%, 52.9 ± 12.8%, and 22.7 ± 2.8%, respectively, relative to the control cells stimulated with LPS only. At the same concentration metabolites **2** and **3** did not show activity in inhibiting the expression of the pro-inflammatory COX-2 expression with LPS treatment, but compound **1** could reduce the expression of COX-2 to 62.8 ± 22.4%. Thus, compounds **1**–**3** might be useful anti-inflammatory agents, while **1** is a promising anti-inflammatory lead compound. Compound **1** could inhibit the expression of both iNOS and COX-2 which might be arisen from the presence of β-hydroperoxy group at C-7 by comparison with compound **2**.

**Table 2:** Cytotoxicity (ED_50_μM) of compounds **1**–**3**.

Compound	Daoy	HEp-2	MCF-7	WiDr
**1**	6.6 ± 0.8	10.4 ±1.1	10.6 ± 0.5	>40
**2**	5.2 ± 0.6	12.3 ± 1.6	10.1 ± 2.3	30.1 ± 2.8
**3**	5.0 ± 0.7	12.4 ± 2.1	6.4 ± 0.5	>40
Mitomycin-C	0.44 ± 0.06	0.30 ± 0.06	0.30 ± 0.12	0.47 ± 0.12

## 3. Experimental Section

### 3.1. General Experimental Procedures

Optical rotations were measured on a JASCO P-1020 polarimeter. Ultraviolet spectra were recorded on a JASCO V-650 spectrophotometer. IR spectra were recorded on a JASCO FT/IR-4100 infrared spectrophotometer. NMR spectra were recorded on a Varian 400MR FT-NMR (or Varian Unity INOVA500 FT-NMR) instrument at 400 MHz (or 500 MHz) for ^1^H and 100 MHz (or 125 MHz) for ^13^C in CDCl_3_. LRMS and HRMS were obtained by ESI on a Bruker APEX II mass spectrometer. Silica gel (Merck, 230–400 mesh) was used for column chromatography. Precoated silica gel plates (Merck, Kieselgel 60 F-254, 0.2 mm) were used for analytical TLC. High-performance liquid chromatography was performed on a Hitachi L-7100 HPLC apparatus with a Merck Hibar Si-60 column (250 × 21 mm, 7 μm) and on a Hitachi L-2455 HPLC apparatus with a Supelco C18 column (250 × 21.2 mm, 5 μm).

### 3.2. Animal Material

*S. crassocaule *(specimen No. 20070402) was collected by hand using scuba off the coast of Dongsha, Taiwan, in April 2007, at a depth of 5–10 m, and stored in a freezer until extraction. A voucher sample was deposited at the Department of Marine Biotechnology and Resources, National Sun Yat-sen University*.*

### 3.3. Extraction and Separation

The frozen bodies of *S. crassocaule *(0.5 kg, wet wt.) were minced and exhaustively extracted with EtOAc (1 L × 5). The EtOAc extract (7.3 g) was chromatographed over silica gel by column chromatography and eluted with EtOAc in *n*-hexane (0–100%, stepwise) then with acetone in EtOAc (50–100%, stepwise) to yield 28 fractions. Fraction 10, eluting with *n*-hexane–EtOAc (6:1), was further purified over silica gel using *n*-hexane-EtOAc (7:1) to afford six subfractions (A1–A6). Subfraction A5 was separated by normal-phase HPLC using CH_2_Cl_2_-Acetone (40:1) to afford **3** (2.1 mg). Fraction 14, eluting with *n*-hexane-EtOAc (3:1), was further purified over silica gel using *n*-hexane-acetone (5:1) to afford eight subfractions (B1–B8). Subfraction B8 was separated by C18 column chromatography and further purified by normal-phase HPLC using CH_2_Cl_2_-Acetone (25:1) to afford **1** (4.6 mg). Fraction 17, eluting with *n*-hexane-EtOAc (1:1), was further purified over silica gel using *n*-hexane-acetone (3:1) to afford seven subfractions (C1–C7). Subfraction C7 was separated by reversed-phase HPLC using MeOH-H_2_O (7:5) to afford **2** (2.2 mg). 

Sarcocrassocolide M (**1**): colorless oil; [α]^25^_D_ −61 (*c* 0.4, CHCl_3_); UV (MeOH) λ_max_ 207 (log ε = 3.6); IR (neat) *v*_max_ 3421, 2961, 2926, 2855, 1757, 1647, 1371 and 1228 cm^–1^; ^13^C and^ 1^H NMR data, see Table 1; ESIMS *m/z* 429 [M + Na]^+^ ;HRESIMS *m/z* 429.1892 [M + Na]^+^ (calcd for C_22_H_30_O_7_Na, 429.1889).

Sarcocrassocolide N (**2**): colorless oil; [α]^25^_D_ −153 (*c* 0.2, CHCl_3_); UV (MeOH) λ_max_ 205 (log ε = 3.5); IR (neat) *v*_max_ 3419, 2927, 1757, 1659, 1434, 1372, 1272 and 1227 cm^–1^; ^13^C and^ 1^H NMR data, see Table 1; ESIMS *m/z* 429 [M + Na]^+^ ; HRESIMS *m/z* 429.1886 [M + Na]^+^ (calcd for C_22_H_30_O_7_Na, 429.1889).

Sarcocrassocolide O (**3**): colorless oil; [α]^25^_D_ −140 (*c* 0.2, CHCl_3_); UV (MeOH) λ_max_ 209 (log ε = 3.7); IR (neat) *v*_max_ 3456, 2970, 2927, 2855, 1752, 1659, 1434, 1381, and 1271 cm^–1^; ^13^C and^ 1^H NMR data, see Table 1; ESIMS *m/z* 371 [M + Na]^+^ ; HRESIMS *m/z* 371.1835 [M + Na]^+^ (calcd for C_20_H_28_O_5_Na, 371.1834).

### 3.4. Cytotoxicity Testing

Cell lines were purchased from the American Type Culture Collection (ATCC). Cytotoxicity assays of compounds **1**–**3** were performed using the MTT [3-(4,5-dimethylthiazol-2-yl)-2,5-diphenyltetrazolium bromide] colorimetric method [26]. 

### 3.5. *In Vitro* Anti-Inflammatory Assay

Macrophage (RAW264.7) cells were purchased from ATCC. *In vitro* anti-inflammatory activities of compounds **1**–**3** were measured by examining the inhibition of lipopolysaccharide (LPS) induced upregulation of iNOS (inducible nitric oxide synthetase) and COX-2 (cyclooxygenase-2) proteins in macrophages cells using western blotting analysis [27].

## 4. Conclusions

Our investigation demonstrated that the soft coral, *S*. *crassocaule*, could be a good source of bioactive substances. The isolated compounds **1**–**3**, in particular **1**, are potentially anti-inflammatory and may become lead compounds in the future drug development. Also, it is noteworthy to mention that cembranoids **1**–**3** possessing an α-methylene-γ-lactonic group with a rarely found 1,1-disubstituted double bond at C-19/C-8 and containing a hydroperoxy group at C-7, were discovered for the first time from corals of this species.
